# PD233 Does Patient Input Add Value To Healthcare Decision-Making? A Two-Year Reflection On Patient Involvement Processes In Singapore

**DOI:** 10.1017/S0266462324004525

**Published:** 2025-01-07

**Authors:** Ping-Tee Tan, Fiona Pearce

## Abstract

**Introduction:**

In 2022, the Agency for Care Effectiveness (ACE) co-developed patient involvement processes with local patient organizations to enable patients and caregivers to share their experiential knowledge about different medical conditions and treatments to inform health technology assessments (HTAs). This presentation describes the impact of patient input on funding recommendations during the first two years of this initiative in Singapore.

**Methods:**

A literature search was conducted to identify frameworks and indicators used by different HTA agencies that could be contextualized to evaluate local patient involvement efforts. Systematic data extraction was performed by two authors to compare patient testimonials received for HTAs conducted between May 2022 and April 2024 with key information in HTA reports, minutes from committee meetings, and published HTA guidance documents to determine the impact of patient and caregiver involvement on the committees’ deliberations and subsequent funding recommendations. The impact of patient involvement on ACE staff, existing processes, and patient participation in future HTAs was also assessed through qualitative surveys.

**Results:**

In the first year (May 2022 to April 2023), 112 patient responses informed 11 HTAs, while 243 responses informed 16 HTAs in the subsequent year (May 2023 to April 2024). At least one testimonial was received for 84.6 percent of HTAs in the first year, increasing to 88.9 percent in the subsequent year. Patient input addressed uncertainties in the scientific evidence and helped decision-making committees understand how different conditions affect patients and their caregivers, the outcomes that matter most to patients, and the benefits and disadvantages of different treatments. In response to feedback, ACE continually evolved its processes to meet the needs of patients and to encourage broader patient participation. Industry and patient organizations also expanded their capacity so that they can meaningfully participate in future ACE HTAs.

**Conclusions:**

Continuous process improvement in response to feedback; providing patient input templates in different formats and languages to improve accessibility; and regular feedback to patient organizations on how their inputs have informed HTAs have increased patient participation, improved the legitimacy of ACE HTAs, and added value to decision-making about which health technologies should be funded in Singapore.

